# Improving Protein Expression Prediction Using Extra Features and Ensemble Averaging

**DOI:** 10.1371/journal.pone.0150369

**Published:** 2016-03-02

**Authors:** Armando Fernandes, Susana Vinga

**Affiliations:** IDMEC, Instituto Superior Técnico, Universidade de Lisboa, Lisboa, Portugal; Massachusetts Institute of Technology, UNITED STATES

## Abstract

The article focus is the improvement of machine learning models capable of predicting protein expression levels based on their codon encoding. Support vector regression (SVR) and partial least squares (PLS) were used to create the models. SVR yields predictions that surpass those of PLS. It is shown that it is possible to improve the models predictive ability by using two more input features, codon identification number and codon count, besides the already used codon bias and minimum free energy. In addition, applying ensemble averaging to the SVR or PLS models also improves the results even further. The present work motivates the test of different ensembles and features with the aim of improving the prediction models whose correlation coefficients are still far from perfect. These results are relevant for the optimization of codon usage and enhancement of protein expression levels in synthetic biology problems.

## Introduction

Metabolic engineering [1], defined as the “directed improvement of product formation or cellular properties through the modification of specific biochemical reactions or the introduction of new genes with the use of recombinant DNA technology” [2], has various industrial applications. It aims at creating microorganisms capable of producing economically relevant chemical products in a cost effective way, which involves optimizing the functioning of metabolic pathways and reactions. These pathways and reactions are controlled by proteins that are frequently heterologous, i.e. external to the organism where they are expressed. This happens, for example, when plant proteins are used to produce chemicals such as vanillin or polyphenols in microorganisms like *Lactococcus lactis* or *Escherichia coli* [3,4]. In order to increase the chemicals production, it is possible to optimise heterologous protein production in the host or target microorganisms [5]. This may be done by altering the codons in the RNA that encodes the protein. Each codon is a sequence of three nucleotides encoding one amino acid that is part of a protein. However, each amino acid can be encoded by several synonymous codons; therefore, a given protein can be expressed using a large number of different codon combinations in the RNA.

The present article proposes to build models, using machine learning algorithms, which are capable of predicting protein expression levels depending on the codon encoding sequence. This allows to test the expression efficiency of a certain protein *in silico* without having to measure it *in vivo*, which is a costly and time-consuming process. One possible reason why the codon sequence determines the amount of protein produced is the influence on the difficulty to unfold the RNA at the cell ribosome at the moment of protein production [6]. Another possible reason has to do with tRNA pools. Since amino acids are attached to tRNA that can only bond to a certain RNA codon, and since the pool of tRNA depends on the microorganism and is larger for certain codons than for others, there might be some difficulty to produce the protein if the RNA employs codons with a poor tRNA pool [7]. Due to this dependence on the organism, the expression levels of the codon encodings employed in the training of the machine learning models must be measured in the destination microorganism.

To the best of the authors knowledge, there are two main works whose focus is on protein expression prediction from codon sequence using machine learning techniques and based on experimental data. They are Welch *et al*. [8] and Supek et Smuk [9], both for *Escherichia coli*. In these two articles the expression levels of various codon encodings were measured and were afterwards used to create expression models. Welch *et al*. created prediction models for two proteins with commercial value, DNA polymerase and a single chain antibody, using partial least squares (PLS). Only codon bias, calculated from codon frequencies, was employed as an input feature. Codon bias measures the tendency of using a certain codon instead of others that encode the same amino acid. The reported validation results were a squared correlation coefficient (R^2^) between the measured and model predicted expression levels of 0.65. Welch *et al*. also demonstrated the poor correlation, of less than 0.01, for Codon Adaptation Index (CAI), a surprising result since CAI is generally thought to be well correlated with protein expression. In the other paper, Supek and Smuc created expression prediction models for a green fluorescent protein (GFP). The models were determined using support vector regression (SVR) and presented a R^2^ between the measured and predicted expression levels of 0.65. The model input features were free energy and codon bias measured as codon frequencies. Free energy is a physical quantity that is minimised as a protein folds. The inclusion of free energy was due to a work by Kudla *et al*. [6] that showed the good correlation of the GFP levels with free energy. Curiously, in Welch *et al*. work there was a small correlation between free energy and protein expression. The fact that there does not seem to be a unique answer on which features correlate the best with protein expression has led the authors of the present article to create prediction models that use more input features than solely codon bias and minimum free energy. This will be shown to improve both the results of Welch *et al*. and Supek et Smuk. In addition, the present article compares two machine learning algorithms PLS and SVR, showing that SVR is better for both Welch *et al*. and Supek et Smuk data. The decision to use the same algorithms as these authors was to show that they were still useful to improve the previously published results when using extra features. Nevertheless, the article goes beyond the isolated use of PLS or SVR models, as in Welch *et al*. and Supek et Smuk works, by improving results with the application of ensemble averaging [10,11].

## Materials and Methods

### Datasets

Two datasets were employed in the present article, one from Welch *et al*. [8] work and the other from Kudla *et al*. [6]. These datasets contain protein expression levels in *Escherichia coli* for different codon sequences. Welch *et al*. dataset contains the expression levels of two proteins, a DNA polymerase and a single chain antibody, for 55 different codon encodings. Kudla *et al*. dataset contains the level of green fluorescent protein produced for 154 different codon encodings. Supek and Smuk [9] uses Kudla *et al*. dataset to create prediction models of protein expression. Both datasets were made available with the articles.

### Features description

**Fig 1:**
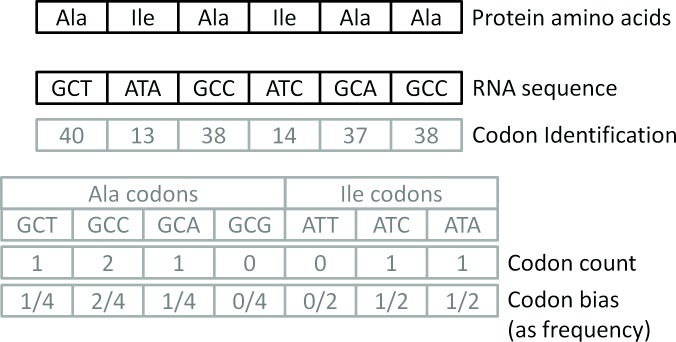
Illustration of the codon bias, codon identification number and codon count features.

The models for protein expression prediction require input features. The ones used were codon bias, codon identification number, codon count and minimum free energy of folding. The first three features are illustrated in Fig 1. Even though the scope of the present article is limited to the analysis of these four features, many others such as the presence of rare codons [12] or the location of G-quadruplexes [13] could have also been used. Codon bias is the difference in the percentage of times (frequency) a certain codon appears in a protein RNA relatively to the total number of codons encoding a certain amino acid. It is estimated by the codon frequency or by the Codon Adaptation Index (CAI) which is a measure of codon bias toward codons frequently used in highly expressed proteins in a certain genome [14]. The present article uses the former. Throughout the article, when "selected codon bias" is mentioned it means that the calculations were done employing the bias of only a subset of codons equal to that from Welch *et al*. article. "Codon bias" only, means that the bias of all codons was used. Codon identification is a number from one to 64 that identifies a certain sequence of three nucleotides that encode an amino acid. Using standard dummy variables for codon identification was not considered to be a good option. In these variables, each codon would be encoded by a vector with 64 entries, 63 of which would have the value zero, and only the position designated for a certain codon would have the value one. Consequently, the vector encodings would be orthogonal [15]. The reason not to use dummy variables is that the instances dimensionality would be unnecessarily large, which could be counterproductive to the effort of giving regression models the ability to provide good results for new inputs, i.e. to generalize. In addition, codon identification numbering (see Table 1) is done in such a way that AAA is closer to AAC than to ACC, because they are more similar, or AAA is closer to CCC than to GGG or TTT, following a rule employed in the construction of a 4-ary tree that reveals hidden symmetries in the genetic code [16]. The rule consists of constructing the tree starting with the sequence A, C, G, T [17]. In fact, the sorting from Table 1 is strongly related to the codon table generated from the 4-ary tree which might explain why this sorting provided better results than random identifications.

**Table 1:** Codon identification numbers.

aaa	1	aac	2	aag	3	aat	4	aca	5	acc	6	acg	7	act	8
aga	9	agc	10	agg	11	agt	12	ata	13	atc	14	atg	15	att	16
caa	17	cac	18	cag	19	cat	20	cca	21	ccc	22	ccg	23	cct	24
cga	25	cgc	26	cgg	27	cgt	28	cta	29	ctc	30	ctg	31	ctt	32
gaa	33	gac	34	gag	35	gat	36	gca	37	gcc	38	gcg	39	gct	40
gga	41	ggc	42	ggg	43	ggt	44	gta	45	gtc	46	gtg	47	gtt	48
taa	49	tac	50	tag	51	tat	52	tca	53	tcc	54	tcg	55	tct	56
tga	57	tgc	58	tgg	59	tgt	60	tta	61	ttc	62	ttg	63	ttt	64

Codon count is the number of times that each codon appears in the RNA sequence. Free energy, also known as Gibbs free energy or minimum free energy (MFE), is minimised as a protein folds so that it reaches a configuration that is as stable as possible. MFE values for the whole mRNA sequence were determined using the Vienna RNA package (http://rna.tbi.univie.ac.at/cgi-bin/RNAfold.cgi) for both Welch *et al*. [8] and Supek and Smuk [9]. MFE values measured between nucleotides -4 and 37 or -4 and 38, for Welch *et al*. and Supek and Smuk, respectively, were obtained together with the datasets from these two works. The nucleotides are counted from the first nucleotide of the first open reading frame (ORF). The use in the models of free energies from the whole protein or from some nucleotides depends on which one provides the best validation results for each dataset.

The features described have dimensionality of one for the MFE and 64 for the codon bias or count. In Welch *et al*., since three codons are missing, the codon bias dimensionality used is only 61. Finally, the codon identification feature has a dimensionality that depends on the protein size, for Welch *et al*. it is 575 and for Supek and Smuk it is 288. Employing all the features simultaneously generates inputs of high dimensionality which poses a risk to good generalization. In total, the features for Welch *et al*. and Supek and Smuk datasets have a dimensionality of 701 and 417, respectively. Good generalization is ensured by the use of the rigorous nested n-fold cross-validation together with partial least squares regression or support vector machines that were designed to handle large dimensionalities. This will be better explained in the next sections.

The rationale for choosing codon bias and minimum free energy as input features is their good results presented in Welch *et al*. [8] and Supek and Smuk [9]. Codon identification number and codon count were selected because the former provides an exact description of the sequence of codons and the latter adds the importance of each codon with respect to the remaining ones in a simple way when compared to codon identification. The features codon bias, codon identification and codon count can be calculated using scripts included in the provided software (please see section "Implementation"). The scripts are inside the directory named “features” of S1 Software, which provides all the code used to generate the results here presented. The MFE must be calculated using the Vienna RNA package.

### Partial Least Squares (PLS) regression

PLS [18] regression is a generalization of multiple linear regression [19] with the advantage that it can handle highly correlated input variables and a number of input variables significantly larger than the number of measurements. PLS combines dimensionality reduction and the creation of the model that transforms the independent into the dependent variables. It works, like principal component analysis (PCA) [20], by creating new variables that correspond to the projection of the independent and dependent variables into new directions. However, these new directions are calculated to maximize the covariance between dependent and independent variables, which is not the case in PCA. The new variables are sorted by the percentage of data variance explained and, typically, the first few are enough to explain most of the data variance. PLS requires adjusting the best number of the new variables called components. This is done by choosing the number of components that yield the smallest validation error.

### Support Vector Regression (SVR)

SVR [21] consists of calculating an approximating function that deviates at most *ε* from the training points and is as flat as possible. This is also called ε-SVR [22]. This approximating function is defined by a set of weights for the input features. Flatness of the function is enforced by maintaining these weights small, which is a form of regularization that helps generalization even with input features of large dimensionality. Since not all points will be inside the *ε* tolerance band, it is necessary to consider slack variables that correspond to the distance from each point to the *ε* band around the approximating function. This function is obtained by solving an optimization problem that consists of minimizing an objective function equal to the quadratic weights size plus the slack variables multiplied by a constant *C*. This constant defines the cost of a point being outside the *ε* band. A constraint that sets the difference between desired values and approximating function to being smaller or equal than *ε* plus the slack variable is added to the minimization problem. The training points inside the *ε* band are not relevant to generate the approximating function, consequently, the training points that generate this function, called support vectors, are sparse. The introduction of kernels further allows to create non-linear functions. These kernels operate by transforming the input points into a feature space where it is easier to create the approximating functions. The ε-SVR used in the present article requires selecting a kernel function and its parameters, as well as the *ε* and *C* values. The kernel used was a radial basis function while the remaining parameters were those providing the smallest validation error.

### Validation method

**Fig 2:**
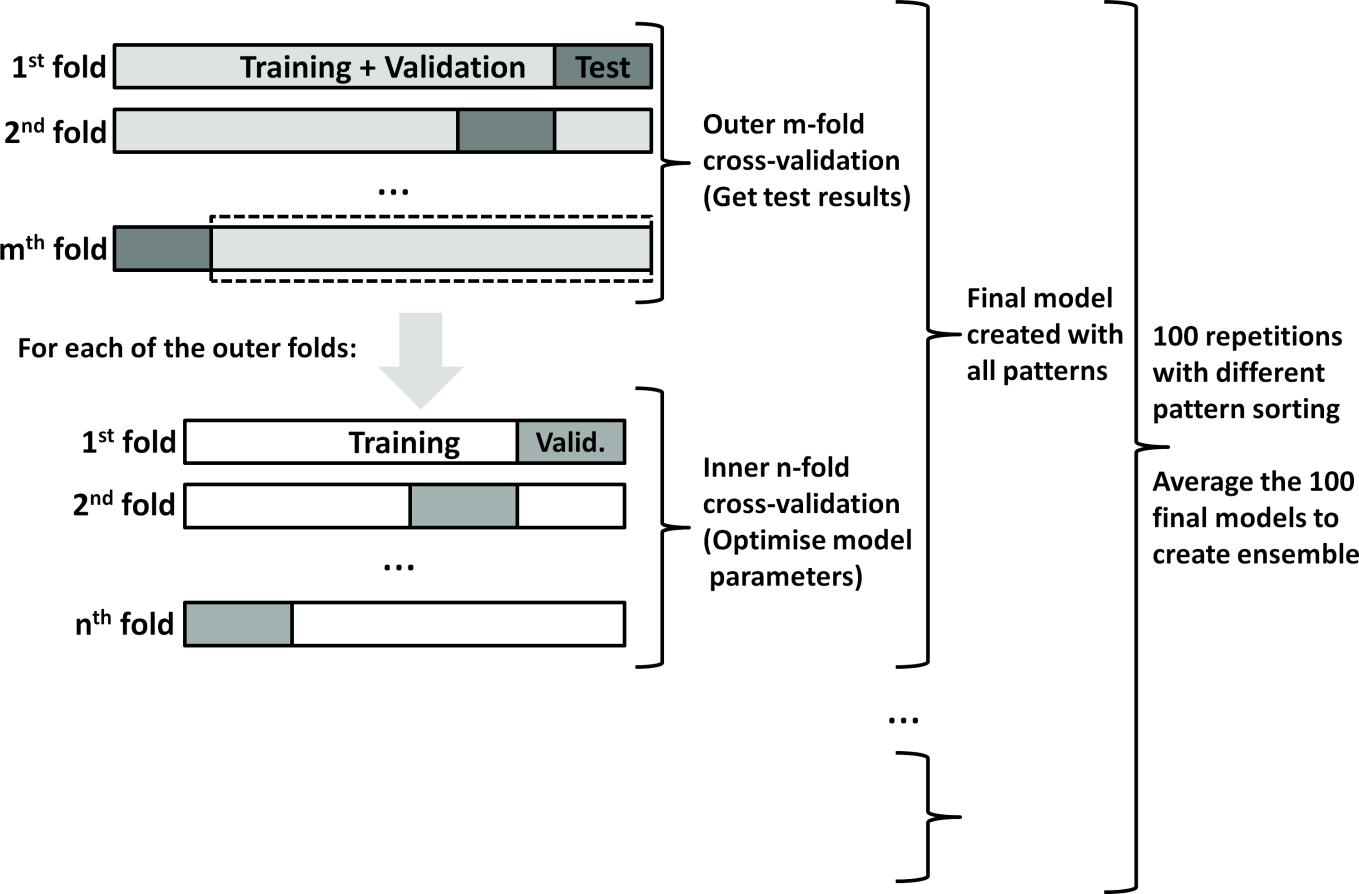
–Schematics of the nested n-fold cross-validation, with repetition and ensemble averaging.

It was necessary to use a validation method to determine which was the best set of parameters and the generalization ability of the created models. The generalization ability is the capacity of the created models to provide correct answers for input patterns not used in the model training. The validation method implemented was nested n-fold cross-validation [23,24] in which there is a n-fold cross-validation inside a m-fold cross-validation, see Fig 2. In n-fold cross-validation the dataset is split into "*n*" folds of equal size and "*n-1*" folds are used for training and one fold for validation. The process is repeated "*n*" times and the overall validation results are the average of the results on each of the "*n*" folds. In nested cross-validation the internal n-fold cross-validation is run for each fold of an outer m-fold cross-validation. The folds of the outer m-fold cross-validation create a test set. In nested cross-validation, the inner n-fold cross-validation provides results that allow to choose a best set of parameters. Once this set is determined, a temporary model is built with all inner folds and used to determine values for data contained in one fold of the outer cross-validation. By repeating the inner cross-validation for all the outer folds and gathering the results for the outer folds it is possible to obtain test results that measure the expected generalization ability of the n-fold cross-validation procedure. The various inner cross-validation may have different sets of parameters. The final model is calculated using all available patterns with the training parameters being obtained by applying n-fold cross-validation to all available patterns without using any patterns for the outer m-fold cross-validation. Nested n-fold cross-validation is thought to provide better estimates for model generalization than simple n-fold cross-validation because the parameter selection is independent from the generalization evaluation [23,24]. However, it also brings additional computational cost.

In the current article, for Welch *et al*. [8] dataset 5- and 11-fold for inner and outer cross-validation were used, respectively. In the case of Supek and Smuk [9] dataset the inner and outer cross-validation was 6- and 7-fold, respectively. The results presented ahead are for the test set and correspond to the average of 100 repetitions of the nested n-fold cross-validation where the pattern grouping in folds is varied.

### Ensemble averaging

Typically, building machine learning models involves creating many versions of the models to determine the best parameters and selecting the model showing the best results. In order not to waste the resources used to create the remaining models it is possible to use ensemble averaging [10,11]. Ensemble averaging consists of making an average of all model results, with the advantage that the ensemble performance is better than that of the best single model. This is so because, as shown theoretically, the bias of a single model and of the ensemble are the same, but, the variance of the ensemble is smaller [10,11]. The model bias measures the difference between the average, over all training sets, of the approximating model and the "real" model. The variance measures the average sensitivity of the approximating model to the training set choice. In the present work, the repetitions in the validation method are necessary because different pattern groupings in the inner n-fold cross-validation lead to different validation results. Therefore, it is possible to do ensemble averaging of the results of various models whose optimal parameters were determined with different pattern groupings. In other words, the ensemble averaging was done using all the 100 repetitions of the nested n-fold cross-validation where the pattern grouping in folds was varied, see Fig 2. The generalization ability for such an ensemble is measured by averaging the test set values from different models for each pattern and afterwards calculating the root mean squared errors (RMSE) and the squared correlation coefficient (R^2^) of the model. It is noteworthy that this is different from averaging the RMSE and the R^2^ of the models from various repetitions as it is done in section "Validation method". The averaging used in the present work gives the same weight to the various models obtained with different pattern groupings.

### Implementation

The necessary software code was written in MATLAB. The PLS implementation used was from MATLAB toolboxes and the SVR algorithm was from LIBSVM [25]. All the code and data is freely available at "http://sels.tecnico.ulisboa.pt/software/" under “Protein expression prediction” and in S1 Software. An example on how to use the created ensembles is given in folder “FinalCodonModel”.

## Results

### Reference calculations

Two models for protein expression prediction were created, one for the dataset from Welch *et al*. [8] and the other for the dataset from Supek and Smuc [9]. The models presented in this section were created with the same algorithms and input features employed in the works of Welch *et al*. and Supek and Smuc. The models here presented are, therefore, an approximate reproduction of the Welch *et al*. and Supek and Smuc works with the difference that the validation method employed in the present proposal, nested n-fold cross-validation, is more complex and should provide more reliable results. The results shown in the present section were used as reference for the improvements in model results that will be presented in the remaining sections. Please notice that the two datasets employed have different values for the expression levels. In Welch *et al*. and Supek and Smuc the expression levels vary between [0.18, 3] and [43, 10606], respectively. Consequently, the RMSE obtained are different. Welch *et al*. employed partial least squares combined with a selection of fifteen codon bias. For this case it was possible to obtain a root mean squared error (RMSE) of 0.570 and a squared correlation coefficient (R^2^) of 0.439. This corresponds to model 1 in Table 2, and, the R^2^ obtained is smaller than the 0.65 reported in Welch *et al*. Supek and Smuc used a support vector machine whose inputs were minimum free energy measured between nucleotides -4 to 37 and codon bias. This led to a RMSE of 1.75e3 and R^2^ of 0.632. These results correspond to model 7 in Table 2, and compare well to the R^2^ of 0.65 reported by Supek and Smuc. The reason why the results were different for Welch *et al*. and similar for Supek and Smuc might be due to the significantly larger number of patterns available for the latter work that makes the change from simple to nested n-fold cross-validation less relevant.

**Table 2:** Test R^2^ and RMSE for various models created using Welch *et al*. and Supec and Smuc datasets. The feature abbreviations are: SelBias—selected codon bias; MFE—minimum free energy; Bias—codon bias; ID—codon identification; Count—codon count.

Dataset	Model	Algorithm	Features	R^2^	RMSE	Observations
Welch *et al*.	1	PLS	SelBias	0.439	0.570	Reference
Welch *et al*.	2	PLS	MFE Bias ID Count	0.487	0.537	-
Welch *et al*.	3	SVR	SelBias	0.427	0.566	-
Welch *et al*.	4	SVR	MFE Bias ID Count	0.504	0.523	-
Supec and Smuc	5	PLS	MFE Bias	0.565	1.91e3	-
Supec and Smuc	6	PLS	MFE Bias ID Count	0.680	1.63e3	-
Supec and Smuc	7	SVR	MFE Bias	0.632	1.75e3	Reference
Supec and Smuc	8	SVR	MFE Bias ID Count	0.698	1.57e3	-

### Model improvement using extra features

#### Welch *et al*. dataset

For Welch *et al*. dataset, model 2 in Table 2 employs PLS, as reference model 1 does, but changes the input features from selected codon bias to an association of minimum free energy for the whole protein, codon bias, codon identification and codon count, improving RMSE to 0.537 and R^2^ to 0.487. Model 3 uses the input features from model 1, selected codon bias, and SVR instead of PLS, achieving a RMSE of only 0.566 and a R^2^ of 0.427. Model 4 consists of SVR combined with minimum free energy between nucleotides -4 to 38, codon bias, codon identification and codon count and obtains the best RMSE of 0.523 and best R^2^ of 0.504.

#### Supek and Smuc dataset

For Supek and Smuc dataset, model 5 using PLS instead of the SVR from reference model 7, but keeping minimum free energy and codon bias as input features presents RMSE of 1.91e3 and R^2^ of 0.565, which is worse than reference model 7. For this dataset the minimum free energy providing the best results for the four models in Table 2 is that between nucleotides -4 and 37. Model 6 uses PLS, as model 5 does, and changes the input features to minimum free energy, codon bias, codon identification and codon count, improving the RMSE to 1.63e3 and the R^2^ to 0.680. Model 8 uses SVR combined with minimum free energy, codon bias, codon identification, and codon count which improves even further the RMSE to 1.57e3 and R^2^ to 0.698.

### Model Comparison

Table 3 and Table 4 present the confidence intervals in the mean difference between two models of Table 2 for a statistical t-test at 5% significance level. If the *p*-value is smaller than 0.05 then the difference in the mean value of the two models is considered to be significant. The models in bold and italic are the ones with best R^2^ and RMSE in the comparison, but the differences may not be statistically significant. The use of t-test has to do with the fact that each mean value is from a distribution of 100 samples originating in the 100 repetitions of the validation method. For this large number of samples the central limit theorem states that the mean will be approximately normally distributed, regardless of the underlying distribution of the samples and, therefore, the t-test can be used. Nevertheless, the authors have conducted the Mann-Whitney test (not shown) and, under this test, the statistically significant results in Table 3 and Table 4 are the same (although with different *p*-values).

**Table 3:** Minimum and maximum values of the confidence intervals for differences in R^2^ at 5% significance level. The situations in bold and italic have the best R^2^. The feature abbreviations are: SelBias—selected codon bias; MFE—minimum free energy; Bias—codon bias; ID—codon identification; Count—codon count. Comp is abbreviation for comparison, M for model and Alg for algorithm.

Dataset	Comp	M	Alg	Features		M	Method	Features	R^2^ difference		
									Min (x10^-2^)	Max (x10^-2^)	p-value
Welch *et al*.	A	***2***	***PLS***	***MFE Bias ID Count***	*vs*	1	PLS	SelBias	3.66	5.92	2.10e-14
Welch *et al*.	B	***4***	***SVR***	***MFE Bias ID Count***	*vs*	3	SVR	SelBias	6.37	8.93	1.60e-24
Welch *et al*.	C	3	SVR	SelBias	*vs*	***1***	***PLS***	***SelBias***	-2.55	0.184	0.0895
Welch *et al*.	D	***4***	***SVR***	***MFE Bias ID Count***	*vs*	2	PLS	MFE Bias ID Count	0.654	2.69	1.41e-3
Welch *et al*.	E	***4***	***SVR***	***MFE Bias ID Count***	*vs*	1	PLS	SelBias	5.22	7.70	4.03e-20
Supec and Smuc	F	***6***	***PLS***	***MFE Bias ID Count***	*vs*	5	PLS	MFE Bias	10.9	12.1	3.08e-94
Supec and Smuc	G	***8***	***SVR***	***MFE Bias ID Count***	*vs*	7	SVR	MFE Bias	5.93	7.22	1.97e-49
Supec and Smuc	H	***7***	***SVR***	***MFE Bias***	*vs*	5	PLS	MFE Bias	6.05	7.28	1.58e-53
Supec and Smuc	I	***8***	***SVR***	***MFE Bias ID Count***	*vs*	6	PLS	MFE Bias ID Count	1.14	2.37	7.72e-08
Supec and Smuc	J	7	SVR	MFE Bias	*vs*	***6***	***PLS***	***MFE Bias ID Count***	-5.41	-4.23	1.67e-37

**Table 4:** Minimum and maximum values of the confidence intervals for differences in RMSE at 5% significance level. The situations in bold and italic have the best RMSE. The feature abbreviations are: SelBias—selected codon bias; MFE—minimum free energy; Bias—codon bias; ID—codon identification; Count—codon count. Comp is abbreviation for comparison, M for model and Alg for algorithm.

Dataset	Comp	M	Alg	Features		M	Method	Features	RMSE difference		
									Min	Max	p-value
Welch *et al*.	A	***2***	***PLS***	***MFE Bias ID Count***	*vs*	1	PLS	SelBias	2.52e-2	3.98e-2	1.45e-15
Welch *et al*.	B	***4***	***SVR***	***MFE Bias ID Count***	*vs*	3	SVR	SelBias	3.55e-2	5.04e-2	3.38e-23
Welch *et al*.	C	3	SVR	SelBias	*vs*	***1***	***PLS***	***SelBias***	-4.33e-3	1.25e-2	0.339
Welch *et al*.	D	***4***	***SVR***	***MFE Bias ID Count***	*vs*	2	PLS	MFE Bias ID Count	8.44e-3	2.06e-2	4.88e-6
Welch *et al*.	E	***4***	***SVR***	***MFE Bias ID Count***	*vs*	1	PLS	SelBias	3.95e-2	5.46e-2	1.23e-25
Supec and Smuc	F	***6***	***PLS***	***MFE Bias ID Count***	*vs*	5	PLS	MFE Bias	266	297	4.46e-90
Supec and Smuc	G	***8***	***SVR***	***MFE Bias ID Count***	*vs*	7	SVR	MFE Bias	155	189	6.37e-49
Supec and Smuc	H	***7***	***SVR***	***MFE Bias***	*vs*	5	PLS	MFE Bias	147	178	4.33e-51
Supec and Smuc	I	***8***	***SVR***	***MFE Bias ID Count***	*vs*	6	PLS	MFE Bias ID Count	36.4	69.9	2.56e-09
Supec and Smuc	J	7	SVR	MFE Bias	*vs*	***6***	***PLS***	***MFE Bias ID Count***	-135	-104	2.76e-34

#### Welch et *al*. dataset

In comparison A (Table 3 and Table 4), we conclude that Model 2 is better than the reference model 1, and the differences in R^2^ and RMSE are both significant. Both models use PLS and different features, with model 2 using more features. In comparison B, both models use SVR with model 4 employing more features, significantly improving model 3. Comparisons A and B suggest that adding input features improves the results, when the algorithm that creates the model is maintained.

In comparison C, the differences between model 3, with SVR, and model 1 that was created with PLS and the same input features are not statistically significant. Nevertheless, comparison D shows that for models 2 and 4 that employ equivalent input features but use PLS and SVR, respectively, the difference in results is statistically significant. Consequently, comparisons C and D indicate, that with Welch *et al*. dataset and equivalent input features, SVR and PLS provide equal results for small number of features but SVR overcomes PLS for larger number of features.

Comparison E shows that model 4 provides the best improvement in the state-of-the-art relatively to reference model 1. The confidence interval for this improvement for Welch *et al*. dataset in R^2^ is [5.22e-2; 7.70e-2].

### Supek and Smuc dataset

In comparisons F and G, the models use the same machine learning algorithm, and the model with more input features wins, suggesting that adding features while maintaining the algorithm allows to improve the results. This is similar to comparisons A and B for Welch *et al*. dataset.

In comparisons H and I where the two model features are the same and the algorithm different, SVR provides better results than PLS, which is similar to comparison D for Welch *et al*. dataset.

In comparison J, PLS which exhibits worse results than SVR when the input features are equal (comparisons H and I), is able to overcome SVR results due to the use of more input features. This indicates that the best models are a combination of the best machine learning algorithms and the best input features, as expected. Comparison G shows an improvement of the state-of-the art of R^2^ for Supek and Smuc dataset, relatively to the reference model, between [0.0593; 0.0722].

### Model improvement using ensemble averaging

**Fig 3:**
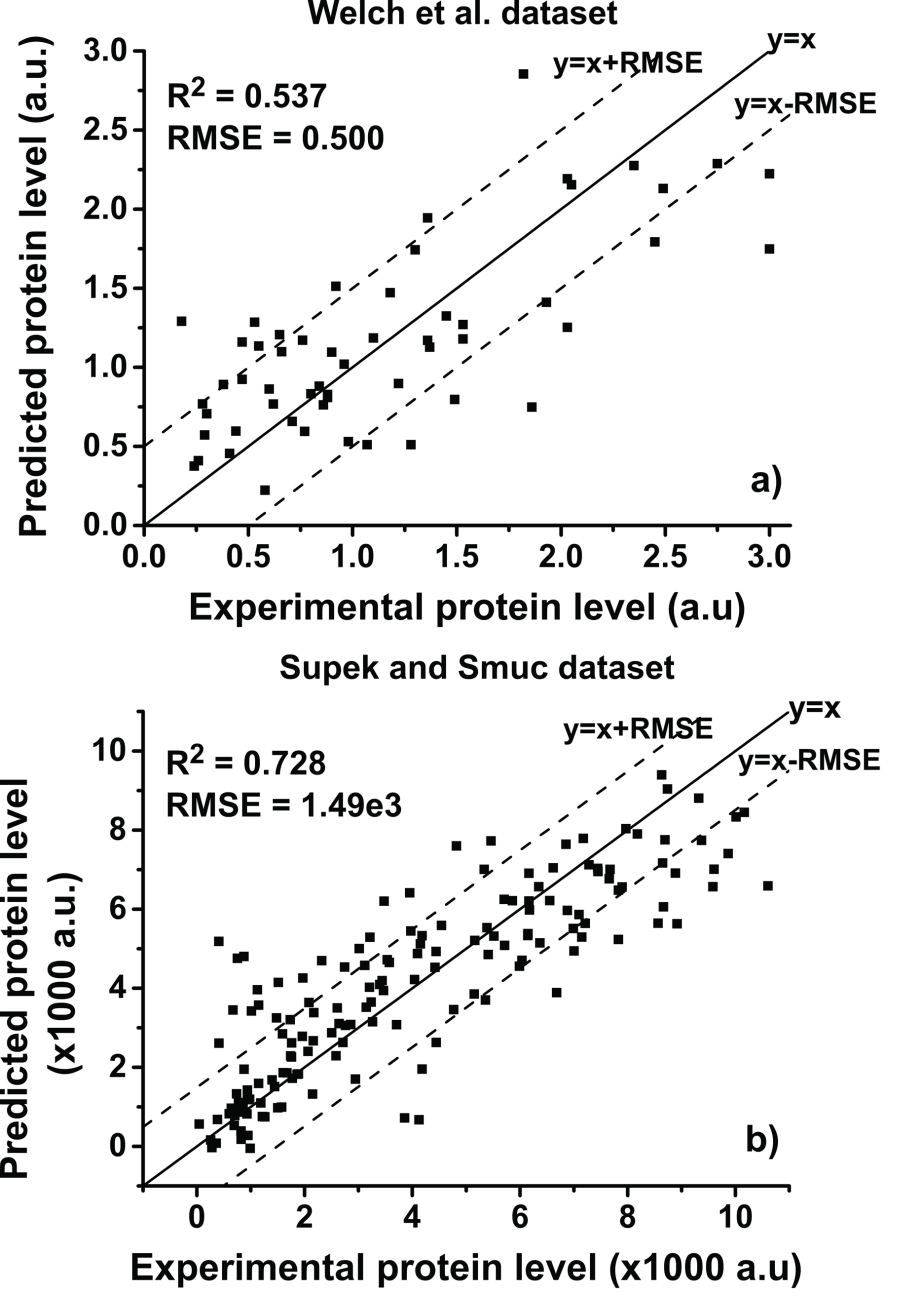
Results for ensemble averaging of validation repetitions.

Even though the state-of-the-art was already improved on the previous sections it can still be enhanced by using ensemble averaging. Fig 3 shows the results of performing ensemble averaging to the 100 repetitions of the validation method. In the case of Welch *et al*. dataset the averaging was done for repetitions of model 4 from Table 2. This way it was possible to improve the RMSE from 0.523 to 0.500 and R2 from 0.504 to 0.537. For Supek and Smuc dataset the ensemble averaging was done with model 8. It allowed to improve the RMSE from 1.57e3 to 1.49e3 and R2 from 0.698 to 0.728.

**Fig 4:**
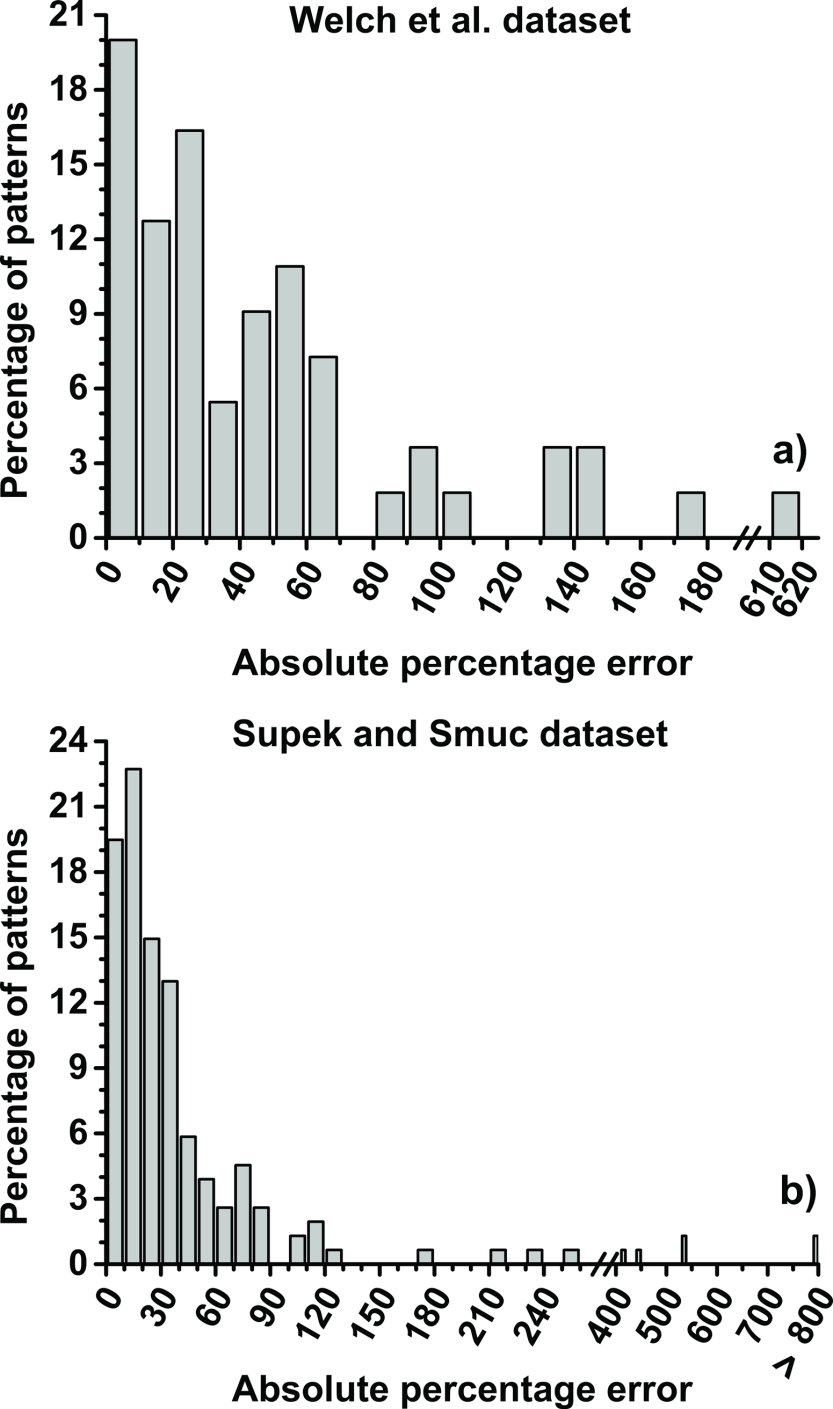
Absolute percentage error for the ensembles from Fig 3.

Fig 4 depicts the absolute percentage error (APE) histograms for the ensembles shown in Fig 3, which allows to quantitatively characterize the distribution of errors from Fig 3. The APE is the absolute value of the difference between the experimental and the predicted protein levels divided by the experimental protein level and multiplied by 100. APE is calculated for each different codon encoding of a protein. In Welch *et al*. dataset, 49.1 and 74.5% of the 55 different codon encoding have an APE smaller than 30 and 60%, respectively. For Supek and Smuc dataset, 57.1 and 79.8% of the 154 different codon encodings have APE smaller than 30 and 60%, respectively. In the distributions in Fig 4 it is possible to see tails of protein encodings with large APE that reduce the R^2^ and increase the RMSE of the models.

## Discussion and Conclusions

The state-of-the-art for the prediction of expression levels of three proteins in *Escherichia coli* has been improved. For the DNA polymerase and the single chain antibody, from Welch *et al*. [8] work, the partial least squares model whose input features were minimum free energy, codon bias, codon identification and codon count followed by ensemble averaging allowed to obtain a R^2^ of 0.537, which improves the 0.439 from partial least squares with selected codon bias from Welch *et al*. work and, the 0.504 from SVR with minimum free energy, codon bias, codon identification and codon count, but no ensemble averaging of model 4 from Table 2. For the green fluorescent protein, from Supek and Smuc [9] work, a support vector regression model whose input was minimum free energy, codon bias, codon identification and codon count and followed by ensemble averaging presented a R^2^ of 0.728. This improved the 0.632 from Supek and Smuc support vector regression with minimum free energy and codon bias and the 0.698 from support vector regression model with minimum free energy, codon bias, codon identification and codon count but no ensemble averaging of model 8 from Table 2. The difference in R^2^ values between Welch *et al*. and Supek and Smuc datasets might originate from the fact that the latter dataset contained almost three times more different codon encodings than the former allowing for more accurate models to be obtained. Another possible reason might be the fact that the former dataset contained two proteins which is a harder problem to handle than the single protein from the latter dataset.

Adding more input features than those employed in the articles of Welch *et al*. and Supek and Smuc clearly improves the predictive ability of the models for protein level estimation. The reason for this is that more input features provide more information which allows for better models to be created. So, even though it is appealing trying to create a simple model based on a single feature, such as pairwise mRNA similarities [26], that is capable of providing good expression level predictions, the results from the present article advise for the combination of any such feature with other in order to improve predictive ability. In fact, the codon identification number feature allows for the machine learning models to have access to the pairwise similarities. The results obtained also suggest that SVR yields better results than PLS for the same input features with Supek and Smuc dataset. A possible explanation for this fact is that PLS creates linear models while SVR models are non-linear having, consequently, a larger processing power. For Welch *et al*. dataset, SVR has also better results than PLS for equivalent input features, but only for the situation with more features, suggesting that for a small number of features SVR nonlinearity might be less relevant. It is important to stress that even when PLS loses to SVR for the same input features, PLS models with more input features can be better than SVR models, showing that a good model is a combination of a good algorithm and a good selection of input features. Ensemble averaging improved the results obtained with extra input features and proper algorithm selection for both the datasets studied.

The models for protein expression prediction developed in the present work are helpful for protein codon optimization. This optimization can be done for a given target protein by submitting to the models several RNA sequences composed of randomly selected synonymous codons and, afterwards, choosing the sequence for which the model indicates the largest protein expression. In a more advanced way, the models can be used to assess the value of the objective function of a genetic algorithm [27] that optimizes the codon sequence. This latter option is employed in Welch *et al*. article [8].

The models created are protein specific and, consequently, the model for Welch *et al*. proteins cannot be used for Supek and Smuc proteins and vice-versa. One reason is that protein expression levels were measured with different scales and there is no way to convert from one scale to the other. In addition, in both datasets, the proteins have different sizes, which changes the codon identification feature and, finally, the minimum free energy was measured for different nucleotides.

Nowadays, there is a market for codon optimization services. Consequently, in spite of the fact that the models for expression level prediction are protein specific, which is not very practical, there are companies willing to develop and improve these models. Partly motivated by possible commercial applications, there is a rich and recent literature in translational kinetics ([12,28–33]). This reveals that a large research effort is currently being made to understand the relation between RNA features and protein expression levels. Even though this problem remains largely unsolved, as the research continues it will be possible to understand better why some features seem to be more relevant for some proteins than for others and it might also be possible to find new features with a large impact on protein expression. This knowledge will lead to a better selection of the input features employed in machine learning models, which are still rarely used for regression problems in protein expression level prediction and, will allow enhancing the proteins codon coding and increasing the heterologous production levels.

## Supporting Information

S1 Software Code and models used to obtain the article results. (ZIP)
